# High-Throughput Sequencing-Based Identification of Arabidopsis miRNAs Induced by *Phytophthora capsici* Infection

**DOI:** 10.3389/fmicb.2020.01094

**Published:** 2020-06-23

**Authors:** Xiaoguo Zhu, Shidan He, Di Fang, Liang Guo, Xiaoyi Zhou, Yushuang Guo, Lei Gao, Yongli Qiao

**Affiliations:** ^1^Shanghai Key Laboratory of Plant Molecular Sciences, College of Life Sciences, Shanghai Normal University, Shanghai, China; ^2^College of Agriculture, Yangtze University, Jingzhou, China; ^3^Laboratory of Molecular Genetics, China National Tobacco Corporation, Guizhou Institute of Tobacco Science, Guiyang, China; ^4^Guangdong Provincial Key Laboratory for Plant Epigenetics, Longhua Bioindustry and Innovation Research Institute, College of Life Sciences and Oceanography, Shenzhen University, Shenzhen, China

**Keywords:** *Arabidopsis*, host-pathogen interaction, high-throughput sequencing, microRNA, *Phytophthora capsici*, target gene

## Abstract

MicroRNAs (miRNAs) are a group of small non-coding endogenous RNAs. In plants, miRNAs play vital functions in regulating growth, development, and stress response. However, the role of miRNAs in *Arabidopsis*-*Phytophthora capsici* (*P. capsici*) model pathosystem is poorly understood. Here, we used a high-throughput sequencing approach to identify pathogen-responsive miRNAs using 15 small RNA (sRNA) libraries prepared from *Arabidopsis thaliana* leaves collected at 0, 3, 6, 12, and 24 h post-inoculation with *P. capsici*. A total of 293 known miRNAs and 6 potential novel sRNAs (miRNAs or siRNAs) were identified, of which 33 miRNAs were differentially expressed at four different infection stages. To verify the reliability of the sRNA-seq results, we investigated the expression of five sRNAs upregulated throughout the four infection stages and their potential target genes using northern blot analysis and/or stem-loop quantitative real-time polymerase chain reaction (qRT-PCR). Gene Ontology (GO) and Kyoto Encyclopedia of Genes and Genomes (KEGG) pathway enrichment analyses revealed that the potential target genes of the differentially expressed miRNAs, both conserved and novel, were enriched in pathways such as starch and sugar metabolism, spliceosome, and plant-pathogen interaction, indicating that the splicing machinery and pathogenesis-related (PR) proteins play important roles in the response to *P. capsici* infection. Taken together, these results provide novel insights into the molecular mechanisms of pathogenesis by *P. capsici*. Additionally, these results will serve as a strong foundation for further in-depth analysis of miRNAs involved in the resistance to *Phytophthora* species in other crops.

## Introduction

MicroRNAs (miRNAs) are a class of endogenous non-coding single-stranded small (21–22 nt) RNAs commonly found in eukaryotes (Reinhart et al., 2002). In plant cells, endogenous *MIR* genes are transcribed to form the primary transcript, pre-miRNA, which is gradually processed in the nucleus in an ATP-dependent manner to first form a pre-miRNA with a hairpin structure and then form an miRNA:miRNA^∗^ double-stranded complex after methylation (Liu et al., 2018). A mature miRNA is produced and combined with a series of proteins to form an RNA-induced silencing complex (RISC), which recognizes target mRNAs for degradation or translation inhibition (Kurihara and Watanabe, 2004). The miRNAs regulate genes at the post-transcriptional level and play an important role in plant metabolism, tissue growth, organ development and differentiation, and programmed cell death (Wightman et al., 1993; Bruscella et al., 2017). Increasing evidence suggests that miRNA is an indispensable regulator of the plant response to biotic and abiotic stresses (Shukla et al., 2008; Zhang et al., 2020).

In plants, miRNAs were first reported as regulators of development and various transcription factors such as MYBs, bZIPs, ARFs, and GRFs (Alonso-Peral et al., 2010; Glazinska et al., 2014; Liebsch and Palatnik, 2020). Recently, it was shown that microRNAs also target other types of genes such as *pathogenesis-related* (*PR*) genes involved in the resistance to invading pathogens (Wang and Galili, 2019). Plant miRNAs change their expression during development and/or in response to environmental challenges. Because of plant-pathogen coevolution, plants have developed two layers of immunity that must be broken by microbial pathogens to cause damage (Dodds and Rathjen, 2010). The first layer of immunity is based on the perception of pathogen-associated molecular patterns (PAMPs) and is known as PAMP-triggered immunity (PTI), which prevents a large number of potential pathogenic microbes from invasion (Jones and Dangl, 2006). The second layer of immunity is known as effector-triggered immunity (ETI). Recent studies demonstrate that small RNAs (sRNAs) are involved in both PTI and ETI in plants (Padmanabhan et al., 2009).

The sRNAs either inhibit gene transcription or degrade mRNAs and participate in the regulation of various physiological plant processes, especially pathogen resistance. For instance, amiR-P69159 and amiR-HC-Pro159, which target the toxicity proteins of the turnip mosaic virus (TuMV) and the turnip yellow mosaic virus (TYMV), induced resistance against TuMV and TYMV infections when expressed in Arabidopsis (Niu et al., 2006). Yin and colleagues showed that treatment of cotton plants with *Verticillium* wilt significantly decreased the expression of miR862 and miR1536 and up-regulated the target gene *TCH4*, indicating that these miRNAs play an important role in defense against *Verticillium* species (Yin et al., 2012). Additionally, overexpression of miR160a and miR398b in transgenic rice enhanced resistance to the fungal pathogen *Magnaporthe oryzae* (Li et al., 2014). In tomatos, miR482 and miR2118 regulate the expression of a nucleotide-binding site leucine-rich repeat (NBS-LRR)-type resistance (*R*) gene (Shivaprasad et al., 2012). Additionally, research shows that miR472 and RNA-dependent RNA polymerase (RDR)-mediated silencing pathways are key regulatory checkpoints that modulate PTI and ETI via post-transcriptional regulation of *R* genes (Boccara et al., 2014).

Oomycetes are a distinct kind of eukaryotic microorganisms that differ from many notorious plant pathogenic fungi, such as *Phytophthora sojae* (*P. sojae*), *P. capsici*, *Phytophthora parasitica*, and *Plasmopara viticola*. Previous reports show that sRNAs in plants are involved in the response to oomycete stress. In soybeans, knocking down the level of mature miR393 led to enhances susceptibility to *P. sojae*, indicating that miR393 promotes defense against *P. sojae* (Wong et al., 2014). Additionally, deep sequencing data revealed the induction of miRNAs in soybeans after an infection with *P. sojae*, indicating these miRNAs impart resistance to *P. sojae* in soybeans (Guo et al., 2011).

In a cucumber, the role of miR164b, miR171e, miR160b, and miR159f was validated in the response to *Pseudoperonospora cubensis* infection (Jin and Wu, 2015). In a tomato, miR1918 was reported to enhance sensitivity to *Phytophthora infestans* infection (Luan et al., 2016). In black pepper, sRNAs derived from the 5’ end of mature tRNAs (5’tRFs) were highly expressed under *P. capsici* stress and targeted defense–related mRNAs, such as *NPR1* (Luan et al., 2016).

*P. capsici* is a soil-borne pathogenic oomycete that causes severe blight and fruit rot of more than 50 plant species, including pepper, tomato, cucumber, and other commercially important crops (Lamour et al., 2012). Blight and fruit rot results in tremendous yield losses approximating $1 billion worldwide each year (Lamour et al., 2012; Howden et al., 2017). Recent research indicates that the *P. capsica*–Arabidopsis system is a model pathosystem for analyzing a wide range of oomycete-plant interactions (Wang et al., 2013). Exploring host-pathogen interactions is the first step toward enhancing our understanding of the molecular basis of pathogenicity and developing disease management strategies that safeguard food production from *P. capsici* infection. Endogenous sRNAs represent a general regulatory mechanism employed by the plant immune system to respond to various pathogens. However, the effect of *P. capsici* infection on endogenous sRNAs of Arabidopsis has not yet been reported.

Here, we constructed and sequenced 15 sRNA libraries prepared from the leaves of *Arabidopsis thaliana* ecotype Columbia (Col-0) at different stages of infection post-inoculation with *P. capsici*. Potential novel and previously known miRNAs were identified in 15 sRNA-seq libraries via bioinformatics analyses. Among the detected miRNAs, 5 up-regulated miRNAs and their target genes were chosen for further examination by northern blot and quantitative real-time PCR (qRT-PCR) analyses. Additionally, we predicted and analyzed potential target genes of the differentially expressed miRNAs by bioinformatics analysis. This study provides useful information for uncovering the regulatory functions of Arabidopsis miRNAs upon *P. capsici* infection and understanding host-pathogen interactions.

## Materials and Methods

### Plant Material and Treatments

Plants of *Arabidopsis thaliana* ecotype Columbia (Col-0) were grown at 22°C in soil in an environmentally controlled growth room under long-day photoperiod (16 h light/8 h dark). Arabidopsis ecotype Col-0 was used in this study because it is highly susceptible to *P. capsici* isolate LT263. The abaxial leaf surface of 3-week-old Col-0 plants was inoculated with 10 μL of *P. capsici* suspension (1 × 10^5^ zoospores mL^–1^) or treated with MgCl_2_ (control). Leaves were collected at 0 (control), 3, 6, 12, and 24 hpi and frozen in liquid nitrogen for RNA extraction. For each sample, 40 leaves of *Arabidopsis* inoculated with *P. capsici* were collected to extract the total RNA. Three independent biological replicates were performed for each treatment.

### *P. capsici* Zoospores Preparation for Plant Infection Assays

*P. capsici* isolate LT263 was cultured on 20% (v/v) V8 agar plates in the dark at 25°C for 4 days (Wang et al., 2013). Cut into uniformly sized hypha pieces (Ø 1.0 cm), incubating mycelial pieces in 10% (v/v) cleared V8 juice in the dark at 25°C for 2 days. Thereafter, the V8 medium was replaced with sterilized water and changed the wash solution once an hour and refresh up to 4 times. After 2 days of incubation under continuous light, many sporangia formed. A zoospore release was induced with a cold shock by placing plates at 4°C for 30 min. The resulting zoospore solution was examined with a light microscope, and then adjusted to the desired concentration with distilled water.

### RNA Isolation, Library Construction, and sRNA-Seq

Total RNA was isolated from *P. capsica*–infected and uninfected Col-0 leaves using the plant RNA reagent (Invitrogen, Life Technologies, United States). The quantity and quality of the isolated total RNA were assessed using a NanoDrop OneC Spectrophotometer. Then, sRNA libraries were constructed using the Small RNA Sample Prep Kit (Illumina, San Diego, CA, United States) and sequenced on the Illumina HiSeq platform by Novogene, Beijing, China.

### Analysis of sRNA-Seq Data

The sRNA reads were mapped to the *Arabidopsis thaliana*_Ensembl_42 genomes^1^ and *P. capsici* genomes that we have sequenced before (unpublished). Reads mapped to the reference sequence were compared with the specified sequence range in miRBase to obtain the details of the matched sRNA in each sample, including the secondary structure, sequence, and length of the miRNA and the number of occurrences of the miRNA in each sample. No mismatch was allowed when the reads were mapped to the genomes. Digestion by the Dicer enzyme converts a miRNA precursor into mature miRNA. Because of the specificity of the digestion site, the first base of the mature miRNA is highly biased (Tang, 2005). Different AGOs recruit its specific subset of small RNAs, such as AGO2 and AGO4 harbors miRNAs that favor a 5′ terminal adenosine, whereas AGO1 preferentially recruit small RNAs with a 5′ terminal uridine (Mi et al., 2008). Therefore, in this study, the base distribution of the first nucleotide of miRNAs of different lengths was analyzed, and the base distribution statistics of each site of miRNAs was also determined.

The signature hairpin structure of potential novel miRNA precursors was predicted using miREvo and mirdeep2 software (Friedlander et al., 2012; Wen et al., 2012). Subsequently, the potential novel sRNAs were further analyzed by strict filtering as described previously (Axtell and Meyers, 2018; Kozomara et al., 2019). The basic principle of miRNA hairpin structure prediction is to analyze the reference sequence of a certain length of sRNA alignment and its secondary structure, Dicer digestion site, energy, and other characteristics. The input data of the miRNA differential expression is read count data obtained from the miRNA expression analysis. Samples with biological duplication were calculated using DESeq2, based on negative binomial distribution. Venn diagrams were constructed using the Calculate online tool^2^. Heatmaps were constructed by using the RStudio software.

### Histological Staining and Microscopy

*P. capsica*–infected Col-0 leaves sampled at 3, 6, 12, and 24 hpi were stained as described previously (Wang et al., 2013). Briefly, the inoculated leaves were fixed in trypan blue (Sigma, St. Louis, MO, United States) for visualizing the infected hyphae. The stained samples were cleared in saturated chloral hydrate until the leaf tissue became translucent. Differential interference contrast (DIC) images were captured using a Nikon 90i microscope (Nikon, Amstelveen, Netherlands).

### Northern Blot Analysis

Total RNA was isolated from *P. capsici*–infected and control Col-0 leaf samples using the TRIzol reagent (Invitrogen, United States). The quality and concentration of total RNA were determined by denaturing gel electrophoresis and NanDrop ND 100x. Northern blot analysis was conducted as described previously (Qiao et al., 2015). Briefly, approximately 20 μg of the total RNA of each sample was analyzed on a denaturing 19% polyacrylamide gel and transferred to Hybond-NX nylon membranes (GE Healthcare, Madison, WI, United States), which were subsequently crosslinked using a Stratagene UV Crosslinker. DNA oligonucleotides complementary to different sequences of miRNAs were synthesized and labeled with biotin (TaKaRa). The membranes were prehybridized with PerfectHyb (Sigma) hybridization solution and then hybridized with the labeled probes. After several washes, the membranes were autoradiographed using a Gel imaging system (Amersham Imager 600, GE, Japan). *U6* RNA was used a loading control. Probe sequences used for northern blot hybridizations are listed in Supplementary Table 1.

### Expression Analysis of miRNAs and Their Targets by qRT-PCR and Stem-Loop PCR

Samples were analyzed by qRT-PCR as described previously (Zhang et al., 2019; Xu et al., 2020). Briefly, 1 μg of total RNA was reverse transcribed using the TransScript II One-Step gDNA Removal and cDNA Synthesis SuperMix (TransGen Biotech). The reverse transcription products were used as templates for qRT-PCR, which was performed on a LightCycler480 II Real-Time PCR System (Roche diagnostics, Mannheim, Germany) using SYBR Premix Taq (TransGen Biotech). *ACTIN1* and *U6* were used as an internal control. The primers used for qRT-PCR and stem-loop RT-qPCR are listed in Supplementary Table 1.

### Prediction and Functional Annotation of miRNA Target Genes

Potential target genes of the differentially expressed miRNAs were predicted using the plant specific-TargetFinder software. To determine the biological function of target genes, GO enrichment analysis was performed using GOseq, which is based Wallenius non-central hyper-geometric distribution (Cai et al., 2006), and KEGG enrichment analysis was performed using the KEGG database^3^ (Kanehisa et al., 2008), which provides genomic, chemical, and systemic information of target genes (Mao et al., 2005). KOBAS software was employed for determining statistically significant enrichment of target genes among the KEGG pathways.

## Results

### Analysis of Compatible Interactions Between Arabidopsis and *P. capsici*

**FIGURE 1:**
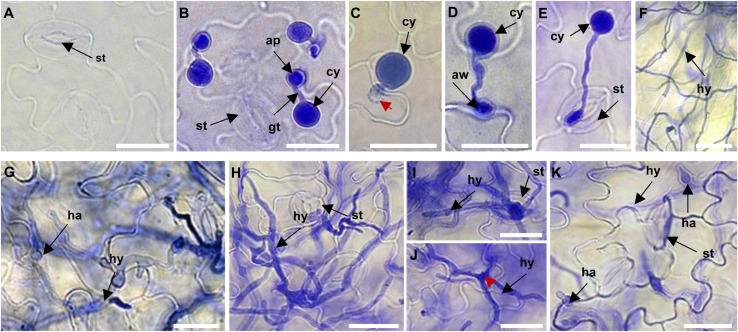
Microscopic analysis of the progression of *Phytophthora capsici* strain LT263 infection in *Arabidopsis thaliana* Col-0 leaves. **(A)** Surface of control leaves not infected by *P. capsici*. **(B)** Germination of zoospores and formation of appressoria on the leaf surface at 3 h post-inoculation (hpi). **(C–E)** Appressorium-mediated penetration of the leaf directly through the epidermis **(C)**, anticlinal cell wall junction **(D)**, or stomata **(E)**. **(F)** Hyphae on Col-0 leaves at 6 hpi. **(F–H)** Massive invasive hyphae with haustoria in the leaf tissue at 6 hpi **(F)**, 12 hpi **(G)**, and 24 hpi **(H)**. **(I,J)** Infection hyphae emerging from the leaves via stomata **(I)** or epidermis **(J)**. **(K)** Haustoria development on the leaf surface. st, stomata; ap, appressorium; gt, germ tube; cy, cyst; aw, anticlinal cell wall junction; ha, haustorium; hy, invasive hyphae. Scale bars = 20 μm.

To identify miRNAs most likely involved in Arabidopsis immunity against *P. capsici*, we performed a thorough evaluation of the infection process of *P. capsici* isolate LT263 in Arabidopsis Col-0 leaves; Col-0 was selected for this experiment because it is highly susceptible to *P. capsici* (Wang et al., 2013). Three-week-old Col-0 leaves were inoculated with *P. capsici* strain LT263 zoospores, and the progression of infection was observed under a microscope. Leaves treated with MgCl_2_ were used as a control (Figure 1A). In leaves infected with *P. capsici* isolate LT263, most zoospores produced germ tubes and attached to the leaf surface at 3 h post-inoculation (hpi), and appressoria were formed at the tips of germ tubes (Figure 1B). *P. capsici* penetrated epidermal cells directly (Figure 1C), entering the leaf tissue either through the junction between epidermal cell walls (Figure 1D) or via stomatal cavities (Figure 1E). At 6 hpi, the hyphae progressed into the adjacent epidermal cells or mesophyll cell layers upon penetration (Figure 1F). Subsequently, the number of haustoria formed from the infection hyphae increased from 12 hpi onward (Figure 1G). At 24 hpi, the inoculated leaf tissues were covered with ramifying mycelia (Figures 1H–K). Taken together, these data suggest that the inoculated Col-0 leaves represented different stages of *P. capsici* infection. Therefore, we used these leaf samples at different infection stages for subsequent library construction and sequencing of sRNAs.

### Deep-Sequencing of sRNA Libraries

To explore the role of miRNAs in *P. capsici* infection, we constructed 15 sRNA libraries from Arabidopsis Col-0 leaves inoculated with *P. capsici*. A total of 16,545,182, 14,799,285, 14,717,539, 15,401,465, and 18,270,638 clean reads were obtained from the inoculated leaf samples collected at 0, 3, 6, 12, and 24 hpi, respectively, which were then mapped on to the Arabidopsis genome (Supplementary Table 2). After filtering to remove tRNAs, rRNAs, small nucleolar RNAs (snRNAs), and other categories of RNAs, we obtained 419,422, 303,754, 445,221, 210,813, and 89,988 reads from known miRNA and 4,928, 3,653, 5,311, 2,718, and 1,180 potential novel miRNA reads from 0, 3, 6, 12, and 24 hpi samples, respectively, ranging in size from 18 to 30 nt (Table 1).

**TABLE 1:** Classification of small RNAs (sRNAs) induced by *Phytophthora capsici* infection in Arabidopsis at different time points.

**Category**	**Sampling time points**				
	**0 h**	**3 h**	**6 h**	**12 h**	**24 h**
Total^a^	8,796,359	6,948,394	7,160,647	6,537,296	7,753,693
Known_miRNA^b^	419,422	303,754	445,221	210,813	89,988
rRNA^c^	3,238,869	2,424,033	2,289,062	2,383,856	3,669,538
tRNA^d^	689,876	533,666	436,080	762,740	883,362
snRNA^e^	16,018	12,326	153,40	10,547	14,738
snoRNA^f^	128,975	130,327	138,232	82,613	20,811
Repeat	409,605	372,075	430,047	234,959	74,160
NAT^g^	107,707	92,122	114,190	64,837	55,908
Novel_miRNA^h^	4928	3653	5311	2718	1180
TAS^i^	12	20	18	6	3
Exon: + ^j^	451,155	396,566	393,982	339,293	250,506
Exon:-^k^	96,492	81,245	97,423	54,713	26,104
Intron: + ^l^	29,626	24,295	23,301	17,251	4557
Intron:-^m^	6963	6697	7576	3684	1113
Other^n^	3,196,710	2,567,615	2,764,864	2,369,264	2,661,724

**^*a*^Number of sRNAs aligned to the reference sequence in each sample; this value was used as a reference for calculating the proportion of various types of sRNAs. ^*b*^Number and proportion of sRNAs in each sample compared with known miRNAs. ^*c,d,e,f*^Number and proportion of sRNAs compared with rRNA, tRNA, snRNA, snoRNA, respectively, in each sample. ^*g*^Number and proportion of sRNAs compared with natural antisense transcripts in each sample. ^*h*^Number and proportion of sRNAs compared with the potential novel miRNA in each sample. ^*i*^Number and proportion of sRNAs compared with the trans-acting siRNAs in each sample. ^*j,k,l,m*^Refer to the number and proportion of positive and negative strands of a sample compared with an exon or intron. ^*n*^Number and proportion of sRNAs in each sample aligned to the reference sequence but not to the known miRNAs, non-coding RNAs (ncRNAs), NAT, novel potential miRNAs, TAS, exons, or introns.**

**FIGURE 2:**
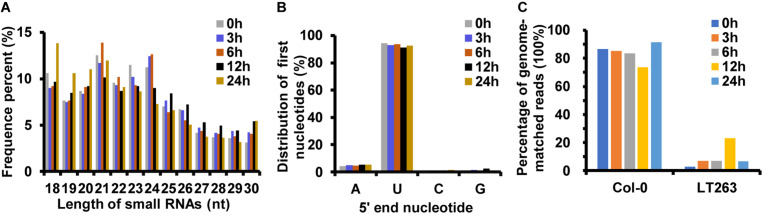
Analysis of small RNAs (sRNAs) by deep sequencing sRNA libraries of Arabidopsis leaves inoculated with *P. capsici*. **(A)** Length distribution of unique mapping reads obtained from 15 sRNA-seq libraries. **(B)** Nucleotide frequency of the 5′ end of previously known sRNAs. **(C)** Percent identity of sRNAs with *A. thaliana* and *P. capsici* genomes.

Analysis of miRNA sequences indicated that 21 and 24 nt reads represented the two most abundant classes of sRNAs at the four infection stages (Figure 2A). More than 90% of the first nucleotide of the 21 and 24 nt miRNA sequence reads was an uracil (Figure 2B) indicating a high consistency in the distribution of plant sRNAs. In the 0, 3, 6, 12, and 24 hpi libraries, 86.73, 85.15, 83.60, 73.63, and 91.55% of the reads mapped on to the Col-0 genome, respectively (Figure 2C). In addition, 6.74, 6.98, 22.92, and 6.40% of the reads from samples collected at 3, 6, 12, and 24 hpi mapped on to the *P. capsici* genome, whereas only 2.95% of the reads from the control sample mapped on to the *P. capsici* genome (Figure 2C). These data indicated that the identified miRNAs were obtained mainly from Col-0. A total of 293 known miRNAs belonging to different families were identified from the 15 sRNA libraries (Supplementary Tables 3, 4). Moreover, 26 unknown sRNAs were predicted based on miREvo and mirdeep2 analyses and secondary hairpin structure prediction (Friedlander et al., 2012; Wen et al., 2012). After strict filtering of unknown sRNAs, we finally identified 6 novel sRNAs, which represented potential novel miRNAs or siRNAs (Table 2 and Supplementary Table 5). To further examine these 6 unknown sRNAs, we investigated whether the flanking sequences of miRNAs could be folded into a signature hairpin structure and exactly mapped to the unknown genome sequence of the plant miRNAs. The predicted hairpin structures of the unknown miRNA precursors showed negative minimum free energies (MFEs), ranging from -27.3 to -116 kcal/mol (average: 62.39 kcal/mol), which was similar to the MFE values of Arabidopsis miRNA precursors (-59.5 kcal/mol) (Bonnet et al., 2004). Additionally, these miRNAs were 21–24 nt in length (Table 2 and Supplementary Figure 1), which is consistent with the typical length distribution of miRNAs (Voinnet, 2009), indicating that these 6 newly identified miRNAs represent potential novel miRNAs.

**TABLE 2:** Potential novel miRNAs identified by sRNA-seq analysis of Arabidopsis leaves inoculated with *P. capsici*.

**miRNA**	**Mature sequence**	**LM**	**Arm**	**LP**	**G + C (%)**	**MFE**
Novel_24	ACCAGUCAACCAUAGAGUCUC	21	5p	284	30.3	−116
Novel_24*	AGACUCUAUGUAGACUGGACU	21	3p	284	30.3	−116
Novel_40	AAAGGACAGAUUACAAGAUACGUG	24	5p	295	41.7	–70.92
Novel_40*	AUAUCAAAUUGGAUCUGUUGUUUC	24	3p	295	41.7	–70.92
Novel_47	AUUUGAUGAACUCGCAAUUAGACG	24	5p	145	34.5	–36.4
Novel_47*	GUUAAUUGCGAGUCGAGAGAAUGA	24	3p	145	34.5	–36.4
Novel_50	UUGUCAUAUCUUGUACCUUCA	21	3p	259	28.6	–88.53
Novel_50*	AAGGCACAAUAUAUGGCAAUG	21	5p	259	28.6	–88.53
Novel_69	UAUGGUUUGAAACUUUGCUUC	21	3p	78	29.5	–35.2
Novel_69*	AGCAAAGUUUCAAACCAUAUU	21	5p	78	29.5	–35.2
Novel_74	AUUAUGAUCAGUUUUUAGACAAGC	24	5p	76	31.6	–27.3
Novel_74*	CCAAAAACUGACCAUAACUA	20	3p	76	31.6	–27.3

LM, length of mature miRNA; LP, length of precursor; MFE, minimal folding free energy. *Represents an RNA sequence of approximately 22 nucleotides that is complementary to miRNA during processing and maturation.

### Expression Profiles of miRNAs in Response to *P. capsici* Infection

**FIGURE 3:**
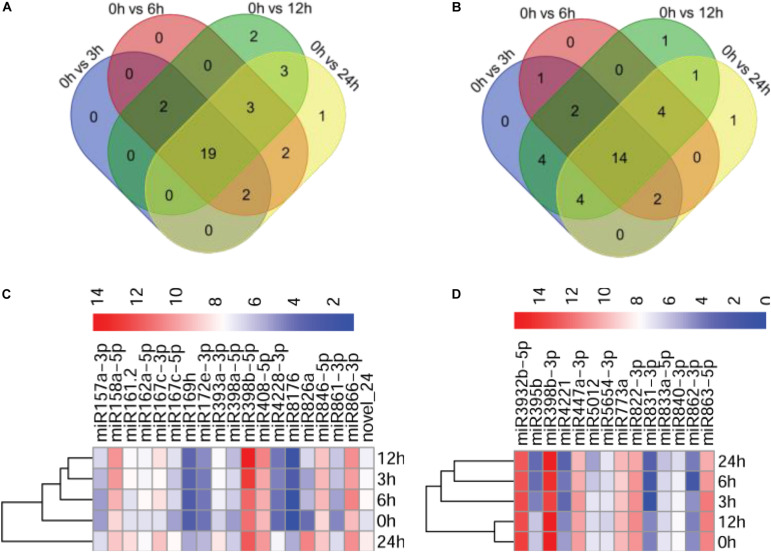
Analysis of differentially expressed miRNAs. **(A,B)** Venn diagrams showing up-regulated **(A)** and downregulated **(B)** miRNAs in *P. capsici*-infected Col-0 leaves at four different infection stages. **(C,D)** Heatmaps of up-regulated **(C)** and downregulated **(D)** miRNAs at four different infection stages. The change in expression, expressed as log2 (TPM + 1), is quantified from high (red) to low (blue), as shown in the color scale.

To identify miRNAs potentially involved in the immunity of Arabidopsis against *P. capsici*, we compared the expression profiles of miRNAs at different infection stages by analyzing high-throughput sequencing data. The sequenced reads that mapped to miRNAs were normalized using the DESeq package, which identified miRNAs showing differential expression among the different infection stages and the control. After normalization, the reads of the tags of each miRNA family were determined as reads per million (*p* < 0.05). The number of miRNA reads generated from the control and *P. capsici*–infected Col-0 samples ranged from several hundreds to several thousands, showing the variability of miRNA transcript abundance. Compared with the control (0 hpi), 23, 28, 29, and 30 miRNAs were up-regulated at 3, 6, 12, and 24 hpi, respectively, and 27, 23, 30, and 26 miRNAs were downregulated at these four infection stages (Figures 3A,B). Among these, 19 and 14 miRNAs were up-regulated and downregulated, respectively, at all four infection stages, as shown by the Venn diagrams (Figures 3A,B) indicating that approximately 30.9% of the newly identified miRNAs were continuously expressed throughout the *P. capsici* infection period.

Next, we investigated the abundance of 19 known upregulated miRNAs exhibiting more than 2-fold (|log2 ratio| ≥ 1) higher expression in at least at one of the four infection stages compared with the control (Supplementary Figure 2). Notably, a potentially novel miRNA, novel_24, was up-regulated in all stages of *P. capsici* infection, suggesting that novel_24 plays an important role in host–pathogen interaction (Supplementary Figure 2A). Similarly, |log2 ratio| < −1 was used as a threshold for selecting downregulated miRNAs. The expression of 14 known miRNAs was downregulated at the four infection stages compared with the control (Supplementary Figure 2B). The heatmap showing the expression patterns of 19 up-regulated miRNAs and 14 downregulated miRNAs at the four different infection stages is shown in Figures 3C,D.

### Validation of Differentially Expressed miRNAs and Their Target Genes

**FIGURE 4:**
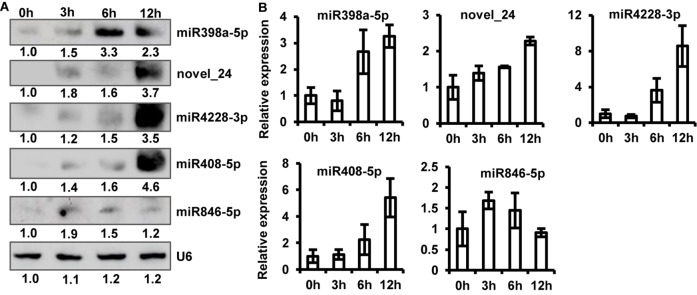
Verification of sRNA-seq data. **(A)** Northern blot analysis of five up-regulated miRNAs at 0, 3, 6, and 12 hpi. *U6* RNA was used as a loading control. **(B)** Relative expression levels of five up-regulated miRNAs analyzed by stem-loop quantitative real-time PCR (qRT-PCR). Transcript abundance of miRNAs was normalized to *U6* snRNA. Data represent mean ± standard error (SE) of three biological replicates.

To validate sRNA-seq results, we examined the expression of five up-regulated (one potential novel and four known miRNAs, including miR398a-5p, novel_24, miR4228-3p, miR408-5p, and miR846-5p, by northern blot analysis. The results of sRNA northern blot analysis were generally consistent with sRNA-seq data. Similar to the sRNA-seq data, the accumulation of all five up-regulated miRNAs was significantly induced to a higher level upon *P. capsici* infection, as shown by northern blot analysis (Figure 4A). However, some discrepancies were observed in miRNA expression levels between sRNA-seq data and northern blot analysis; for example, unlike sRNA-seq analysis, the northern blot assay showed greater accumulation of novel_24, miR4228-3p, and miR408-5p at 12 hpi than at other time points (Figure 4A).

To further confirm this observation, we performed stem-loop qRT-PCR and quantified the abundance of miRNA transcripts. Stem-loop primers were designed to amplify the biologically active form of miRNAs (pri-miRNAs). Expression profiles of qRT-PCR products are shown in Figure 4B. The results of stem-loop qRT-PCR were consistent with those of sRNA-seq and northern blotting analyses. The transcript abundance of novel_24, miR4228-3p, and miR408-5p was the highest at 12 hpi, while that of miRNA846-5p was the highest at 3 hpi compared with the control (Figure 4B). Thus, the stem-loop qRT-PCR directly confirmed that sRNA-seq data effectively identified differentially expressed miRNAs of Arabidopsis induced by *P. capsici*.

**FIGURE 5:**
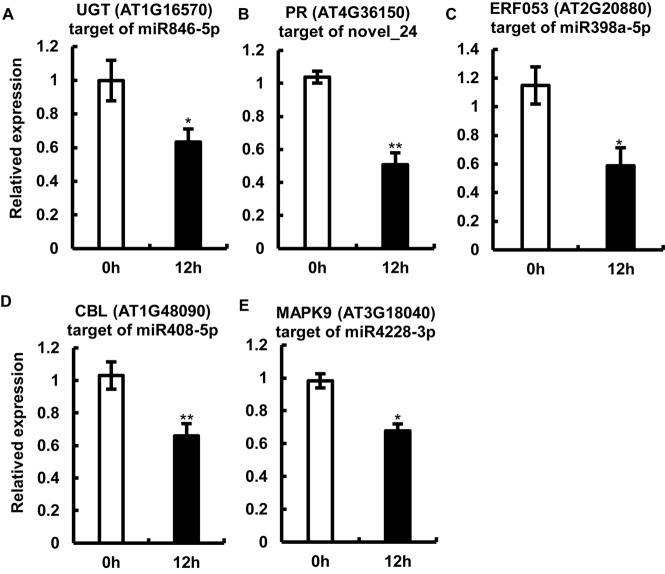
Expression analysis of miRNA target genes by quantitative real-time RT-PCR. **(A–F)** Relative expression levels of *AT1G16570* (target of miR846-5p) **(A)**, *AT4G36150* (target of the potential novel miRNA, novel_24) **(B)**, *AT2G20880* (target of miR398a-5p) **(C)**, *AT1G48090* (target of miR408-5p) **(D)**, and *AT3G18040* (target of miR4228-3p) **(E)** in Col-0 leaves at 12 hpi. Arabidopsis leaves treated with MgCl_2_ were used as a control. The *Actin* gene was used as an internal control for data normalization. Data represent mean ± SE of three biological replicates. Single or double asterisks indicate significant differences (*p* < 0.05 or *p* < 0.01, respectively).

Because the miRNAs regulate gene expression by cleaving target mRNAs and suppressing mRNA transcription (Krol et al., 2010; Wang and Galili, 2019), we examined the transcript abundance of one of the potential candidate targets of each of the five miRNAs by qRT-PCR (Figure 5). The expression of the tested mRNAs showed a negative correlation with the abundance of miRNAs, which is consistent with one of the roles of miRNAs (Figure 5). Interestingly, sequences of the target gene and its cognate miRNA showed highly complementary (Supplementary Figure 3). Among the tested target genes, three genes including *AT3G18040*, *AT1G48090*, and *AT1G16570* showed a drastic reduction in expression (approximately 60%) upon *P. capsici* infection (Figure 5); *AT3G18040* encodes mitogen-activated protein kinases (MAPKs, e.g., MAPK9, one of the targets of miR4228-3p), *AT1G48090* encodes calcium-dependent lipid-binding (CBL) protein (target of miR408-5p), and *AT1G16570* encodes a putative UDP-glycosyltransferase (UGT; target of miR846-5p). Additionally, the expression of genes encoding a drought-inducible transcription factor ERF053 (*AT2G20880*; target of miR398a-5p) and TIR-NBS-LRR-type PR protein (*AT4G36150*; PR, target of novel_24), was repressed in Col-0 leaves by approximately 50% at 12 hpi. These target genes are likely involved in the regulation of Arabidopsis immunity against *P. capsici*. Collectively, these results showed a clear negative correlation between the expression levels of target mRNAs and their corresponding miRNAs, thus providing key insights into miRNA-mediated gene regulation under pathogen stress.

### Functional Annotation and Signaling Pathway Analysis of Potential Target Genes

**FIGURE 6:**
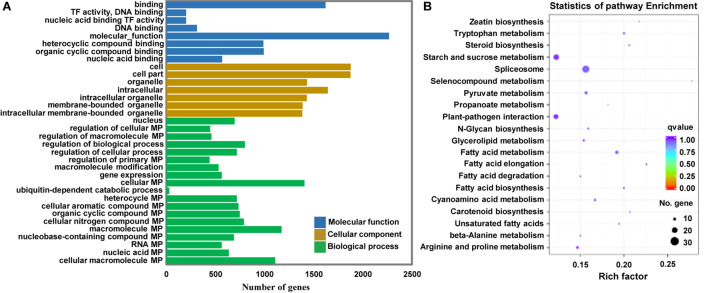
Functional analyses of target genes of the differentially expressed miRNAs at 0, 3, 6, 12, and 24 hpi. **(A)** Gene ontology (GO) enrichment analysis. The molecular function, cellular component, and biological process GO categories are indicated in blue, orange, and green, respectively. **(B)** Kyoto Encyclopedia of Genes and Genomes (KEGG) pathway analysis. The *q*-values varied from 0 to 1, as shown in the color scale. The size of the dot indicates the number of genes in the corresponding pathway. TF, Transcription factor; MP, Metabolic process. Rich factor, numbers of enriched genes/numbers of background pathway genes.

Because miRNAs identify target genes via sequence complementarity, identification of the target transcripts and potential functions of miRNAs is essential for a comprehensive understanding of miRNA-mediated gene regulation. To further investigate the possible role of the identified miRNAs in regulating Arabidopsis immunity, we first predicted the potential target genes of miRNAs in the Arabidopsis database using TargetFinder. Subsequently, we performed Gene Ontology (GO) and Kyoto Encyclopedia of Genes and Genomes (KEGG) enrichment analyses of the target genes of miRNAs differentially expressed at 12 hpi. A total of 31,427 potential targets of miRNAs were assigned to 34 categories (Figure 6A). A detailed summary of the GO classification is shown in Figure 6A. In the cell component category, the most abundant terms were cell, cell part, and intracellular. In the molecular function category, the most abundant GO terms were binding and molecular function. In the biological process category, the metabolic process and regulation of biological process showed the highest enrichment. KEGG pathway analysis revealed that 20 pathways were enriched in response to *P. capsici* infection including spliceosome and plant-pathogen interaction (Figure 6B). Thus, the results of functional annotation indicated that *P. capsici* regulates the immunity of Arabidopsis via differentially expressed miRNAs.

## Discussion

Plant endogenous miRNAs, a class of small non-coding RNAs, play vital functions in host-pathogen interactions. Identification of miRNAs showing differential expression in response to pathogen infection is the first step toward the elucidation of their functions in plant immunity. Previously, several studies identified a set of pathogen-induced miRNAs, and functionally characterized their role in plant immunity (Navarro et al., 2006; Du et al., 2011; Radwan et al., 2011; Seo et al., 2013; Li et al., 2014). However, the effect of *P. capsici* infection on Arabidopsis Col-0 miRNAs has not been reported to date.

In this study, we first investigated the infection process of *P. capsici* by microscopic evaluation, which showed clear correlation between the time from inoculation and *P. capsici* infection stages (Figure 1). This finding is consistent with a previous study on Arabidopsis (Wang et al., 2013), which contributed to the determination of sampling time points and subsequent functional analysis. In the current study, 333 miRNAs differentially expressed in response to *P. capsici* infection were identified by deep sequencing, and most of these miRNAs were highly conserved. Seemingly, the number of known miRNAs that negatively regulate Arabidopsis immunity is greater than that of positive regulators. All 293 known miRNAs belonged to 176 families. The number of miRNAs in each family varied greatly, with MIR156, MIR167, MIR169, MIR172, MIR398, and MIR396 families containing the most members. The expression of different miRNAs also varied greatly (Supplementary Table 4); miRNA826a was the most highly expressed, which is similar to the expression of the nitrogen (N) starvation-induced miR826 (Liang et al., 2013), while miR395b showed the lowest expression, which is consistent with a previous study showing the induction of miR395 in Arabidopsis under sulfate starvation conditions (Jones-Rhoades and Bartel, 2004).

The objective of this study was to identify miRNAs up-regulated in Arabidopsis upon *P. capsici* infection. We examined five up-regulated miRNAs including four known miRNAs (miR398a-5p, miR408-5p, miR846-5p, and miR4228-3p) and one potential novel miRNA (novel_24). All five miRNAs were up-regulated as soon as *P. capsici* zoospores attached to the leaf surface, and their expression remained up-regulated throughout the infection process. Although some studies previously reported the role of miR846-5p and miR398a-5p in plant biotic stress response (Xie et al., 2018), no study has investigated the sRNAs roles of *Phytophthora* species about these five miRNAs between *Phytophthora* species and host plants. In soybeans, knocking down the level of mature miR393 enhances susceptibility to *P. sojae* (Wong et al., 2014). Our sRNA-seq data showed that miR393-3p and miR169h were also induced upon *P. capsici* infection. Furthermore, we verified the transcript abundance of all five differentially expressed miRNAs by RNA blot and qRT-PCR analyses. The results suggested that our sRNA-seq data are highly reliable, and the identified Col-0 miRNAs were up-regulated in response to *P. capsici* infection.

Increasing evidence shows that miRNAs are highly conserved across species and cleave the same or similar target genes. Such as miR156, miR165/166, miR167, miR169, miR171, and miR172, they target crucial transcription factors belonging to the AP2, ARF, bZIP, and WRKY families in multiple species (Sire et al., 2009; Lee et al., 2010). These target genes play the most basic role in regulating plant growth, development, and biotic and abiotic stress responses (Wang and Galili, 2019). In this study, the predicted target genes of known miRNAs, such as miR398a-5p and miR408-5p, were conserved among different plant species and targeted common factors such as resistance related genes. Given the interaction of known miRNAs with their common targets in different plant species, these known miRNAs likely employ similar mechanisms to regulate *P. capsici*–triggered immunity in Arabidopsis. The miR398a-5p is conserved across different plant species and targets *ERF* genes, which regulate disease resistance pathways (Gutterson and Reuber, 2004; Meng et al., 2013; Dong et al., 2015). Consistent with the induction of miR398a-5p, we detected reduced expression of *ERF053* genes in *P. capsici*–infected leaves. The repression of *ERF053* genes may contribute to the repression of *PR* genes, thus affecting plant defense. In wheat, miR408 targets *TaCLP1*, which promotes resistance against stripe rust (Feng et al., 2013). In this study, the gene encoding calcineurin B-like (CBL)-interacting protein kinase, chosen as the potential target of miR408-5p in Arabidopsis, was downregulated in *P. capsici*–infected leaves, implying that miR408-5p negatively regulates plant immunity by affecting the CBL-interacting protein kinase (CIPK)-CBL signaling pathway, which is involved in the response to various biotic and abiotic stresses (Xi et al., 2017; Aslam et al., 2019). In this investigation, while miR846-5p was up-regulated, its target gene, *UGT*, was downregulated in Col-0 leaves. Considering that *UGT* genes promote plant immunity, suppressing the expression of *UGT* genes may inhibit the response of Col-0 to *P. capsici* infection. Some of the known but non-conserved miRNAs (such as miR4228-3p), which were also detected in the present study, have been identified only in one or a few plant species so far. The expression of *AT3G18040* (one of the targets of miR4228-3p), was significantly downregulated in Col-0 leaves, which may inhibit the MAPK signaling pathway. Notably, accumulation of one potential novel miRNA was detected in Arabidopsis leaves inoculated with *P. capsici*. Nevertheless, consistent with the up-regulation of novel_24, its target *PR* genes were downregulated. PR proteins are generally induced by different types of pathogens, such as fungi, oomycetes, and viruses (van Loon et al., 2006). Because PR proteins accumulate at the infection site in response to the invading pathogen and contribute to systemic acquired resistance (SAR) (van Loon et al., 2006), downregulation of *PR* genes by the novel_24 in response to *P. capsici* infection possibly repressed PTI in *Arabidopsis*. In plant-microbe compatible interactions system, there seem to be more miRNAs that negatively regulate plant immunity than those that do positively (Li et al., 2014). Similarly, during the Arabidopsis–*P. capsici* interaction system, the plant also can employ its own miRNAs to downregulate genes that are important for defense. We speculate that the pathogen could suppresses miRNA biogenesis or directed manipulation of host miRNA during infection process in a compatible interaction system. Thus, how miRNAs induced due to pathogen infection or the pathogen-derived miRNAs and their biological implications are need to be explored. A northern blot assay confirmed the increased abundance of these miRNAs, including miR398a-5p, miR408-5p, miR846-5p, miR4228-3p, and novel_24, in infected plants. Further investigation is needed to determine how miRNAs interact with their target genes and how the latter are expressed under biotic stress conditions. Thus, future studies will need to focus on the functional investigation of miRNA target genes and identification of functional components of the regulatory network.

## Data Availability Statement

All datasets generated for this study are included in the article/Supplementary Material. The sRNA deep sequencing data has been deposited in the Sequence Read Archive (SRA) database with BioProject accession number in NCBI is PRJNA607881.

## Author Contributions

YQ conceived and designed the experiments. XGZ, SH, LiG, LeG, XYZ, and DF performed the experiments. XGZ analyzed the experiment data. YQ and XGZ wrote the manuscript. All authors read and approved the final manuscript.

## Conflict of Interest

The authors declare that the research was conducted in the absence of any commercial or financial relationships that could be construed as a potential conflict of interest.

## Footnotes

1 ftp://ftp.ensemblgenomes.org/pub/plants/release-42/fasta/arabidopsis_thaliana/dna/

2 http://bioinformatics.psb.ugent.be/webtools/Venn/

3 http://www.genome.jp/kegg/
