# Supplementary material for: High-Throughput Sequencing-Based Identification of Arabidopsis miRNAs Induced by Phytophthora capsici Infection
Source: Front Microbiol. 2020 Jun 23;11:1094. doi: 10.3389/fmicb.2020.01094 (PMC7324540; doi:10.3389/fmicb.2020.01094)
Supplement: Supplementary file 2 [file Data_Sheet_1.PDF]

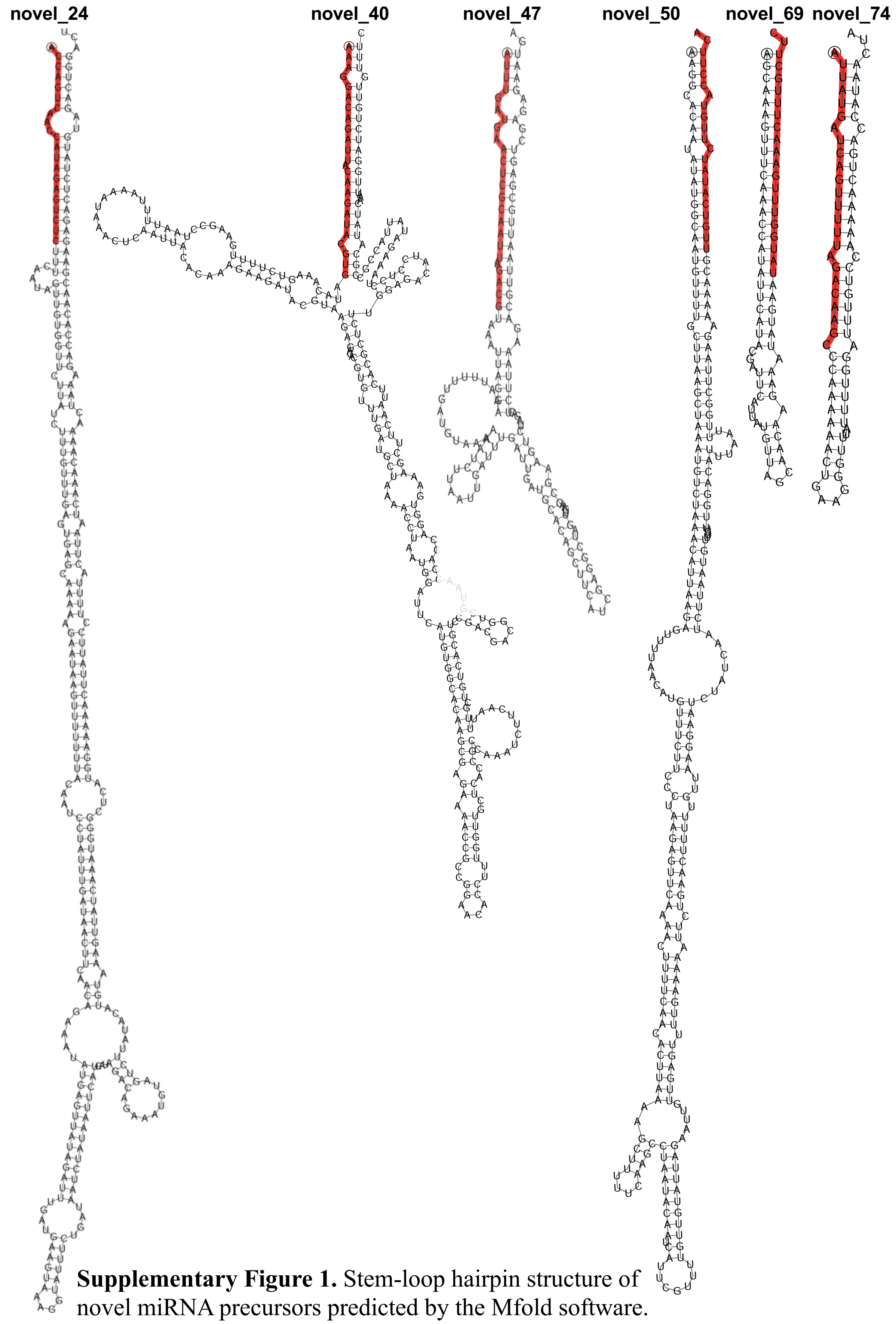

Supplementary Figure 1. Stem-loop hairpin structure of novel miRNA precursors predicted by the Mfold software.

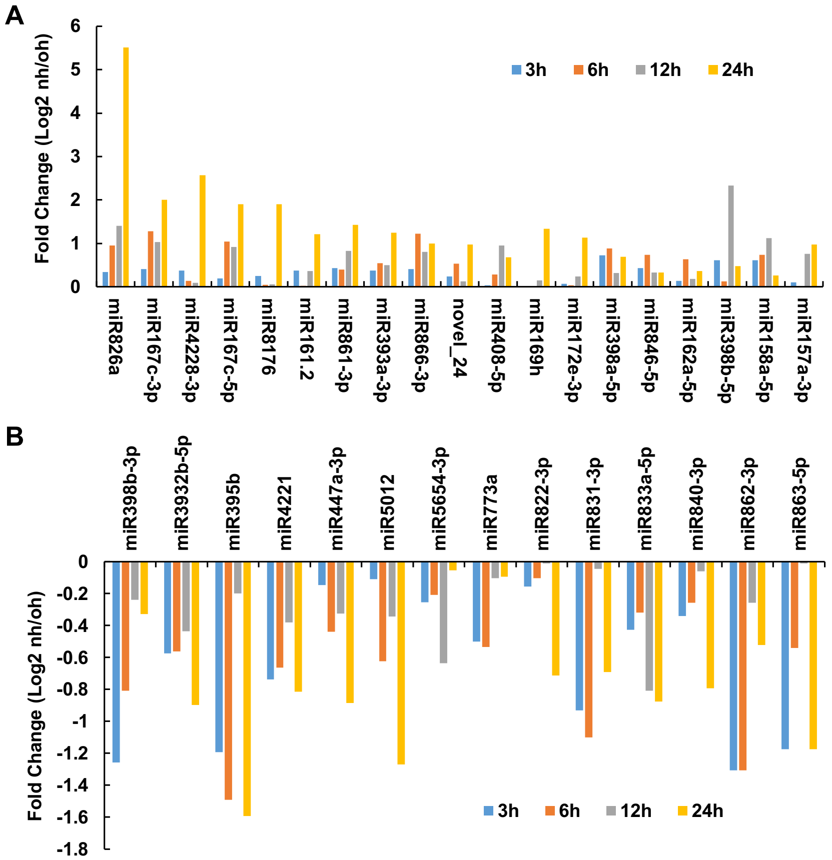

Supplementary Figure 2. Expression profiles of miRNAs identified by small RNA sequencing (sRNA-seq) of *Phytophthora capsici*-infected *Arabidopsis thaliana* Col-0 leaves at four different infection stages. (A) Up-regulated miRNAs. (B) Down-regulated miRNAs.

```
miR408-5p           21 GUACGAGACGAACAAGGACA 1
                        :| :|||||| |||:|||||
AT1G48090-CBL     7029 AGUUUUCUGCUGGUUUCCUGU 7049

miR4228-3p          21 UGUGGUGGCAAAGCGUAGGCU 1
                       :||||::| | |:||||::||
AT3G18040-MAPK9    873 GCACCGUCUUCUUGCAUUUGA 893

miR846-5p           21 GACUUAUCUUCAGGAACUUAC 1
                       || ::| |||||||| |:|||
AT1G16570-UGT       43 CUUGGUCGAAGUCCUCGGAUG 63

novel_24            21 CUCUGAGAUACCAACUGACCA 1
                       |||:: ||: | |||||||||
AT4G36150-PR      1096 GAGGUCCUGAGAUUGACUGGU 1116

miR398a-5p          21 ACACAAGUGUACGGUGAGGAA 1
                       | | ||| :||||:||||||
AT2G20880-ERF053   221 UAUCUUCUUAUGCUACUCCUC 241
```

Supplementary Figure 3. Predicted targets of known and novel miRNAs induced by *P. capsici* infection.
